# ASO Author Reflections: Total Neoadjuvant Therapy with Chemotherapy Alone for Pancreatic Cancer?

**DOI:** 10.1245/s10434-024-15660-8

**Published:** 2024-06-22

**Authors:** Phoebe N. Miller, Eric K. Nakakura

**Affiliations:** 1 Division of Surgical Oncology, Section of Hepatopancreaticobiliary Surgery, Department of Surgery, University of California, San Francisco, San Francisco, CA USA; 2 Helen Diller Family Comprehensive Cancer Center, San Francisco, CA USA

## Past

For patients with pancreatic adenocarcinoma (PDAC), surgery followed by multiagent chemotherapy is the most effective treatment.^1,2^ Because many patients are unable to complete total (6 months) adjuvant chemotherapy, neoadjuvant therapy has emerged as an important strategy for all surgical stages: resectable, borderline-resectable, and locally advanced PDAC.^2^ Also, there is growing interest in total neoadjuvant therapy (TNT) combining chemotherapy and radiation therapy.^3^ However, the optimal duration of neoadjuvant treatment and the role of radiation therapy remain undefined.

## Present

Our institutional practice to use long-duration neoadjuvant chemotherapy started for patients with borderline resectable and locally advanced PDAC, and after favorable results, extended to those with resectable disease.^4^ The use of long-duration therapy (median cycles: FOLFIRINOX = 10; gemcitabine-based = 7) is unique.^5^ Some might even consider this TNT consisting of chemotherapy only. Most (85%) patients received FOLFIRINOX without radiation therapy, and the R0 resection rate was 76%. The median OS was 41 months—the same for all surgical stages.

## Future

Although it was not our official policy, patients with all surgical stages generally received neoadjuvant chemotherapy for 6 months unless shortened or stopped because of adverse effects (~25% of patients).^5^ With FOLFIRINOX, stable disease was expected after four cycles, and for approximately half of patients who demonstrated tumor shrinkage, smaller tumors were not usually seen until after eight cycles. For patients with borderline resectable and locally advanced disease that persisted radiographically after neoadjuvant chemotherapy, radiation therapy was used selectively if the CA 19-9 remained elevated. FDG-PET/CT was not regularly used for Lewis antigen-negative patients to guide therapy decisions.

Given these favorable results, future studies of TNT for all stages of PDAC might consider including a systemic therapy alone arm.^5^ Our findings also reinforce that PDAC is a systemic disease requiring good systemic and surgical therapy for favorable outcomes.
